# Regression as Attachment Behavior in Medical Care

**DOI:** 10.1192/j.eurpsy.2026.11056

**Published:** 2026-07-31

**Authors:** E. M. Jetter, D. Valle, H. Nyugen, B. R. Carr

**Affiliations:** 1 College of Medicine; 2 Department of Psychiatry, University of Florida, Gainesville, United States

## Abstract

**Introduction:**

Regression in illness, long described by Anna Freud as a defense mobilized under stress rather than childishness (Freud A. The Ego and the Mechanisms of Defense 1936), can represent a return to earlier modes of coping. Winnicott reframed regression to dependence as a potential opportunity for repair (Winnicott DW. Metapsychological and Clinical Aspects of Regression 1954). In the medical setting, such dynamics often appear as attachment behaviors such as seeking reassurance, resisting care, or withdrawing, and are often misread as resistance. Bowlby situated these responses within the broader framework of attachment and loss (Bowlby J. Attachment and Loss 1969), while Adshead emphasized that psychiatric staff themselves may function as attachment figures, shaping how regression is expressed and understood in treatment (Adshead G. Psychiatric Staff as Attachment Figures 1998).

**Objectives:**

To consider how concepts of attachment and regression help explain patient behavior in medical settings, and how this understanding can improve care.

**Methods:**

A narrative review was conducted of psychoanalytic, attachment, and psychosomatic medicine literature. The review examined how regressive behaviors are described and their reported effects on communication, adherence, and the therapeutic relationship.

**Results:**

When patients regress, their behavior often reflects attachment needs under stress. These responses typically fall into three recognizable patterns: (1) protest/regression, expressed as repeated pleas for help and a wish for closeness and reassurance; (2) anger/resistance, seen as pushback against treatment when needs feel unmet; and (3) withdrawal, an apparent indifference or shutdown that reflects retreat from connection when overwhelmed. Viewed in this way, behaviors that might otherwise be seen as defiance are better understood as coping. Recognizing these patterns helps clinicians respond with steadier boundaries and reassurance instead of frustration.

**Conclusions:**

Understanding regression as attachment-driven reframes difficult behaviors as expected responses to stress. This approach reorients difficult behaviors from resistance to expected responses to stress, giving clinicians concrete strategies for steadier, more empathic care while maintaining boundaries and sustaining trust.

**Disclosure of Interest:**

None Declared

